# On Mercury in Typhus Fever

**Published:** 1808-04

**Authors:** 


### *To the Editors of the Medical and Physical Journal.*

Gentlemen,

THE rage for novelty pervades Medical Society, in common with others; there is a fashion even in medicine; and it would seem, that it matters not, however absurd and preposterous the opinions of some men are, so that they are but new and singular. I am led to this observation from hearing, almost daily, different medicines announced, and highly extolled in the cure of particular complaints, by men high in the medical world, eagerly taken up by others, and, after a trial, as readily abandoned; thus we have seen Digitalis, Uva Ursi, and Lichen Islandicus, Oxydum Bismuthi, Dr. Lambe's distilled water, and a long catalogue of et ceteras, fall into disrepute, and fail in realizing the advantages which we were taught to believe would result from their employment in particular cases, cases, and for which they had been considered as almost infallible. I would not deprecate the spirit of enquiry and experiment, when conducted with moderation, and upon reasonable principles; all I mean to contend for is, that we are not to set those things down for facts to be depended upon in the application of medicine, which are supported only by a case or two of uncertain benefit.

We now have mercury recommended as a cure for typhus fever; and Dr. Mease, in the last Number of the Medical and Physical Journal, appears to expect much good will result from its exhibition in that complaint. The propriety of its employment seems to me very doubtful, and, I must confess, my experience of it does not encourage me to place such confidence in its powers as Dr. M. appears to do; it may very possibly be useful, but, if I might judge from a case which fell under my observation some time since, it would give me reason to suspect it was of probable detriment. The patient, in this instance, was a female of between thirty and forty years of age, and had been attended by a neighbouring practitioner, who had administered mercurial medicine so as to produce its full effect upon the mouth. She laboured under a complete phrenzy; I found her tied down in her bed with cords; her pulse was extremely rapid and feeble; tongue of a dark brown colour; and spitting very copious. I did not ascertain the exact quantity of mercury she had taken; but, as she had had the ptyalism for nine or ten days, it may be presumed the quantity was pretty considerable. She was, at this time, ordered the bark with wine and opium, and spuma cerevisi, and with great difficulty recovered, and that only after a considerable time had elapsed. Now, as there was no abatement of the symptoms when the salivation came on, or during its continuance, I do not think we can reasonably suppose the mercury had any share in effecting the cure; indeed, so far was she from mending during the time she was under its influence, that she progressively became worse.

I know that the medicine in question has been tried extensively, both externally and internally, in a neighbouring sea-port town, where the population is large, and the appearance of typhus pretty constant; and I know likewise that it has not answered the expectations of the medical men in that place, many of whom had placed great reliance upon its supposed power in cutting short this disease.

Every one must be ready to agree with Dr. M. that the insensibility of the system is often such as not to be easily overcome overcome by the usual stimuli; and that, from the length of time required in the treatment of this complaint, it becomes tiresome and expensive, as well as injurious to both the mind and body of the patient, and that any plan which would promise to shorten its duration deserves general adoption; but judging, as I do, of the uncertainty of medical conclusions, I think I am justified in saying we ought not to set our hopes on so precarious a foundation as a remedy, which probably will be found has been recommended too hastily, and which, it is to be suspected, will not prove worthy of the confidence its advocates would repose in it.

*Feb.* 13, 1808. HARLESTONENSIS.

---

### *To the Editors of the Medical and Phyfical Journal.*

GENTLEMEN,

